# 

**Published:** 1902-07

**Authors:** 


### CONTENTS.

ORIGINAL ARTICLES:
A Deviation from the Orthodox Method of Treating Parturient Paresis and Its Etiology. By W. E. A. WYMAN, V.S. . . . 405
President's Address. By P. O. KOTO, M.D.C. . . . 407
Tuberculin. By H. F. PALMER, D.V.S. . . . 410
Obstetrics. By JAMES M. REED, V.S. . . . 416
Phlegmasia Dolens, Or What? By C. MCKIM, M D.C. . . . 419
DEPARTMENT OF CANINE, FELINE, AND AVIAN MEDICINE AND SURGERY . . . 424
ABSTRACTS FROM FOREIGN JOURNALS . . . 428
REPORTS OF CASES:
A Report of Three Interesting Cases. By GEORGE M. BOON, V.S. . . . 433
Impaction of the Fourth Fold of the Large Colon of a Horse, Complicated by Strangulation of the Ileum in the Foramen of Winslow—Fatal. By JOHN J. REPP, V.M.D. . . . 436
Phytolacca Poison in Cattle. By G. R. WHITE, D.V.S. . . . 439
Purpura Hemorrhagica. By C. A. PAISLEY, V.S. . . . 441
EDITORIALS:
Our Frontispiece . . . 442
A.V.M.A. . . . 442
NECROLOGY . . . 444
COMMENCEMENT EXERCISES—AMONG THE COLLEGES—SOCIETY PROCEEDINGS—NOTES—PERSONALS . . . 445

